# Effect of guided self-determination youth intervention integrated into outpatient visits versus treatment as usual on glycemic control and life skills: a randomized clinical trial in adolescents with type 1 diabetes

**DOI:** 10.1186/1745-6215-15-321

**Published:** 2014-08-12

**Authors:** Gitte R Husted, Birger Thorsteinsson, Bente Appel Esbensen, Christian Gluud, Per Winkel, Eva Hommel, Vibeke Zoffmann

**Affiliations:** Department of Pediatrics, Nordsjællands Hospital Hillerød, University of Copenhagen, Dyrehavevej 29, 3400 Hillerød, Denmark; Patient Education Research, Steno Health Promotion Center, Steno Diabetes Center, Niels Steensens Vej 8, 2820 Gentofte, Denmark; Department of Cardiology, Nephrology and Endocrinology, Nordsjællands Hospital Hillerød, University of Copenhagen, Dyrehavevej 29, 3400 Hillerød, Denmark; Research Unit of Nursing and Health Science, Copenhagen University Hospital, Glostrup, Ndr. Ringvej 57, 2600 Glostrup, Denmark; Department of Public Health, Faculty of Health and Medical Sciences, University of Copenhagen, Blegdamsvej 3B, 2200 Copenhagen, Denmark; Copenhagen Trial Unit, Center for Clinical Intervention Research, Department 7812, Rigshospitalet, Copenhagen University Hospital, Blegdamsvej 9, 2100 Copenhagen, Denmark; Steno Diabetes Center, Niels Steensensvej 2, 2820 Gentofte, Denmark; Juliane Marie Centre, Rigshospitalet, Copenhagen University Hospital, Blegdamsvej 9, 2100 Copenhagen, Denmark; Nasjonal kompetansetjeneste for læring og mestring innen helse (NKLMH) Oslo University Hospital, Kierkeveien 166, 0424 Olso, Norway

**Keywords:** Type 1 diabetes mellitus, Adolescents, Outpatient clinic, Hospital, Clinical trials, Randomization, Empowerment

## Abstract

**Background:**

Providing care for adolescents with type 1 diabetes is complex, demanding, and often unsuccessful. Guided self-determination (GSD) is a life skills approach that has been proven effective in caring for adults with type 1 diabetes. To improve care, GSD was revised for adolescents, their parents, and interdisciplinary healthcare providers (HCP) to create GSD-Youth (GSD-Y). We evaluated the impact of GSD-Y after it was integrated into pediatric outpatient visits versus treatment-as-usual, focusing on glycemic control and the development of life skills in adolescents with type 1 diabetes.

**Methods:**

Seventy-one adolescents (mean age: 15 years, mean duration of diabetes: 5.7 years, mean HbA1c: 77 mmol/mol (9.1%), upon entering the study) from two pediatric departments were randomized into a GSD-Y group (n = 37, GSD-Y was provided during individual outpatient sessions) versus a treatment-as-usual group (n = 34). The primary outcome was the HbA1c measurement. The secondary outcomes were life skills development (assessed by self-reported psychometric scales), self-monitored blood glucose levels, and hypo- and hyperglycemic episodes. The analysis followed an intention-to-treat basis.

**Results:**

Fifty-seven adolescents (80%) completed the trial, and 53 (75%) completed a six-month post-treatment follow-up. No significant effect of GSD-Y on the HbA1c could be detected in a mixed-model analysis after adjusting for the baseline HbA1c levels and the identity of the HCP (*P* = 0.85). GSD-Y significantly reduced the amotivation for diabetes self-management after adjusting for the baseline value (*P* = 0.001). Compared with the control group, the trial completion was prolonged in the GSD-Y group (*P* <0.001), requiring more visits (*P* = 0.05) with a higher rate of non-attendance (*P* = 0.01). GSD-Y parents participated in fewer of the adolescents’ visits (*P* = 0.05) compared with control parents.

**Conclusions:**

Compared with treatment-as-usual, GSD-Y did not improve HbA1c levels, but it did decrease adolescents’ amotivation for diabetes self-management.

**Trial registration:**

ISRCTN 54243636, registered on 10 January 2010. Life skills for adolescents with type 1 diabetes and their parents.

## Background

Managing type 1 diabetes during adolescence is a complex and demanding process that is often unsuccessful [1, 2]. The importance of good glycemic control for preventing or postponing the long-term complications of diabetes has been well established [3]. Although late diabetic complications are rarely observed during adolescence, these pathogeneses begin to develop soon after diagnosis and accelerate during puberty [4]. In adolescents with type 1 diabetes, the target for glycemic control is a HbA1c level of less than 58 mmol/mol (7.5%) and an absence of frequent hypoglycemia [5]. Currently, 31% of adolescents in Denmark achieve this target (overall mean HbA1c: 70 mmol/mol, 8.6%) [6]. This result emphasizes the need for new methods to address the complexity of treating and caring for diabetes during adolescence.

Improvements in glycemic control during adolescence are associated with the involvement of parents and healthcare providers (HCPs) through a constructive and autonomy-supportive relationship that leads to self-determined management of the disease [7]. Behavioral and psychosocial interventions to improve self-management and glycemic control have had only a moderate effect [8], and only a few interventions involving parents have been integrated into conventional outpatient care [9–11].

Guided self-determination (GSD), a life skills approach developed to facilitate empowerment in the patient-provider relationship, has been shown to be effective in group training for adults with type 1 diabetes and persistently poor glycemic control, reducing HbA1c by 3 mmol/mol (0.4%) and increasing their life skills [12]. GSD functions as a shared decision-making and mutual problem-solving method involving the use of semi-structured reflection sheets [13] in combination with mirroring [14], active listening [15], and value-clarifying responses [16], which lead to focused communication and situational reflection [17]. Life skills are defined as ‘those personal, social, cognitive, and physical skills that enable people to control and direct their lives and develop the capacity to live with and produce change in their environment’ [18]. The core principle in GSD is to support patients in clarifying and expressing their difficulties and mobilizing their own potential for change in interactions with autonomy-supportive HCPs. Instead of being instructed by HCPs, GSD guides patients and HCPs in shared decision-making, whereby patients find solutions that align with their own values [17].

We adjusted GSD into GSD for adolescents and their parents (GSD-Youth (GSD-Y)) and fully integrated this method into pediatric diabetes outpatient clinics run by the adolescents’ usual interdisciplinary HCPs [13]. The adjustment and implementation of GSD-Y lasted 18 months. Authors GRH and Zoffmann VZ adjusted the adult GSD to the GSD-Y version in collaboration with 22 adolescents (between 13 and 18-years-old), their parents and nine interdisciplinary HCPs [19, 20], ensuring that the method was suitable for pediatric care in dyads and triads. The adjustment did not alter the original purpose of GSD. Only minor vocabulary changes and a reduction in the number of semi-structured reflection sheets were made to fit this new context.

The aim of our randomized clinical trial was to test whether GSD-Y reduced HbA1c levels and improved life skills in adolescents with type 1 diabetes compared with conventional outpatient diabetes care.

## Methods

### Study design

The study was a randomized clinical trial with a mixed-methods design. The protocol has been previously published [13]. Here, we present the results from the quantitative portion of the trial.

### Participants

Between September 2009 and November 2010, adolescents with type 1 diabetes attending two Danish pediatric outpatient clinics at two hospitals in the Capital Region of Denmark were recruited for the trial if they met the following eligibility criteria: a) aged between 13 and 18-years-old; b) had been diagnosed with type 1 diabetes for more than one year; c) had engaged in insulin therapy since the onset of the disease; d) had recorded levels of HbA1c ≥64 mmol/mol (8.0%) at their last evaluation before entry into the trial (determined from medical records), and had maintained an average HbA1c of >58 mmol/mol (7.5%) during the year prior to inclusion, with values collected from the Danish National Diabetes Register for Children (DanDiabKids) [6] and manual searches of local medical records; e) had not been diagnosed with any psychiatric disease; f) were not engaged in psychological treatment at the time of recruitment; and g) had the ability to speak and understand Danish. Parents were included if they spoke, read, and wrote Danish, did not have severe illnesses, did not have mental health problems, and were not currently undergoing psychiatric or psychological treatment. The criteria for discontinuation from the trial included the voluntary withdrawal of consent or (at the discretion of the investigator) the occurrence of severe concomitant disease or non-compliance with the trial protocol. The criterion for HCPs to participate in the trial was at least one year of experience in a diabetes pediatric outpatient clinic at the beginning of the intervention. The HCPs were GSD-Y trained and tested for their abilities to provide GSD-Y correctly in triads of adolescents, parents, and HCPs prior to the start of the trial [13].

Written informed consent was obtained from all adolescents and parents of minors (younger than 15 years of age) prior to enrollment by the adolescents’ usual HCP. The trial protocol was reviewed by the Danish National Committee on Biomedical Research Ethics in April 2009 as a registry- and interview-based research study (REC; reference number 0903054; document number 230436). The study was registered with the Danish Data Association (reference number 2008-41-2322) and with the Current Controlled Trials registry (reference number ISRCTN54243636).

The adolescents were stratified according to their usual HCPs and were randomized in blocks . A case report form (CRF) was used on the day of randomization to ensure that the adolescents fulfilled the eligibility criteria. The adolescents were randomized using opaque sealed envelopes containing a twice-folded piece of paper indicating the group assignment; these assignments were prepared in blocks of four, each comprising two GSD-Y intervention assignments and two usual-care assignments. The four envelopes in each block were randomly mixed and then consecutively numbered from one to four by GRH. In collaboration with VZ, GRH supervised the HCPs during randomization. Because of the nature of the intervention, neither the adolescents nor the HCPs could possibly be blinded to the group allocation after randomization. All participating adolescents provided a blood sample for HbA1c measurement while in the clinic before the randomization. Baseline characteristics are presented in Table 1.

**Table 1:** **Clinical and demographic baseline characteristics of the adolescents**

	GSD-Y	CONTROL
	37	34
n (% females)	22 (62)	21 (60)
Age (years)	14.9 ± 1.5	14.6 ± 1.3
BMI (kg/m^2^)	22.1 ± 2.9	22.3 ± 4.0
Age at onset of diabetes (years)	8.8 ± 2.9	9.2 ± 3.7
Duration of diabetes (years)	6.1 ± 3.0	5.3 ± 3.4
HbA1c (mmol/mol)	79.9 ± 16.6	72.8 ± 9.4
HbA1c (%)	9.5 ± 3.7	8.8 ± 3.0
SMBG (number per week)	28 ± 14	33 ± 18
Insulin dose (IU per kg per day)	1.2 ± 0.6	1.0 ± 0.5
MIT, n (%)	25 (68)	22 (65)
CSII, n (%)	12 (32)	12 (35)
Living with both parents, n (%)	16 (62)	21 (70)
Ethnicity		
Danish, n (%)	31 (84)	25 (74)
Other, n (%)*	6 (16)	9 (26)
Education		
Danish public school (0-10 grades), n (%)	23 (62)	25 (74)
Secondary education, n (%)**	8 (22)	5 (15)
Other schools, n (%)***	6 (16)	4 (11)

CSII: continuous subcutaneous insulin infusion; GSD-Y: Guided Self-Determination-Youth group; MIT: multiple insulin injections; SMBG: self-monitored blood glucose. Data are presented as means ± SDs (number of patients (%)).

*Turkey, Somalia, Sweden, France, Russia, Morocco, Afghanistan, Poland, Tunisia, Pakistan.

**Gymnasium, Higher Preparatory Examination (HF), Higher Commercial Examination Program (HHX), Higher Technical Examination Program (HTX).

***Continuation school.

### GSD-Y intervention

Two pediatric physicians, five pediatric diabetes nurses, and two dieticians (HCPs) provided the GSD-Y intervention as part of their conventional outpatient clinical care. The intervention was divided into eight sessions scheduled over an 8- to 12-month-period with a standard duration of one hour per session in an individual setting. The intervention consisted of 18 semi-structured reflection sheets for adolescents, five for parents, and six reflection sheets if the adolescent was visiting a dietician [13]. The details of all predefined topics for each session for either adolescents or their parents are published elsewhere [13]. The reflection sheets were given to adolescents and parents in the outpatient clinics prior to each session and they were asked to complete their individual reflection sheets between sessions with regard to the different predefined main topics that related to their lives with diabetes (for example ‘Room for your diabetes’ or ‘Room for your teenager’s diabetes in your life’). By completing the reflection sheets using their own words and drawings, the adolescents and parents systematically explored and prepared to express their individual and shared difficulties with diabetes when coming to outpatient sessions. Each session started with going through the reflection sheets together with HCPs focusing on issues that were accentuated by the participants. The use of mirroring [14], active listening [15], and values-clarifying responses [16] in their communication helped them mutually reflect on the issues. The GSD-Y sessions hereby functioned as a life skills training process [21] in six steps: (1) establishing a mutual relationship with clear ‘I-you’ (a form of interpersonal communication) borders, also called I-you-sorted mutuality [22], (2) self-exploration, (3) self-understanding, (4) shared decision-making, (5) action, and (6) feedback from action.

The adolescents were invited to complete one session (session two) without their parents to facilitate conversation about their confidential personal affairs. The parents could participate in the remainder of their adolescents’ sessions by mutual agreement. To address the parents’ challenges, they were offered two GSD-Y sessions alone with the HCP. Their sessions were scheduled twice during the experimental period (after three and six months), typically lasting one hour per session.

GSD-Y adolescents’ need to see a dietician was determined if the reflection sheets completed after session one or two indicated it. Each referral to a dietician was anticipated to involve a minimum of two sessions in addition to the planned eight sessions. A referral to a dietician could take place during the entire trial period.

The adolescents and parents kept their original semi-structured reflection sheets and copies were placed in the adolescents’ medical records.

### Treatment-as-usual control group

Adolescents in the control group were also offered eight sessions, which were scheduled equal to the intervention group across an 8- to 12-month-period, with a maximum standard duration of 45 minutes (usually 30 to 45 minutes). They received typical outpatient care; measurement of HbA1c, advice on how to improve glycemic control, discussions about whether to change the insulin dose or type of administration. Parents participated as before and, if necessary, a referral to a dietician was made based on the HCPs’ individual judgment because no guidelines were available for this procedure.

### Outcomes

#### Primary outcome

The primary outcomes were HbA1c levels measured at baseline and every third month during the trial. The HbA1c levels from both hospitals were analyzed at the same department of clinical biochemistry using the Variant Analysis Mode of the Tosoh Automated Glycohemoglobin Analyzer HLC-723G8, Alere A/S, Park Allé 350E, 2605 Brøndby, Denmark (normal range 23 to 40 mmol/mol, 4.3 to 5.8%). As the HbA1c analyses in Scandinavia were found to be falsely high due to problems with a freeze-dried calibrator, these values were consequently decreased by 2.7 mmol/mol (0.24%), following recommended guidelines [23].

#### Secondary outcomes

The secondary outcomes consisted of the following six scales that measure the multifaceted process of developing life skills: the five-item Perceived Competence in Diabetes Scale (PCD) measured the degree of competence perceived by patients in managing diabetes [24]; the five-item Health Care Climate Questionnaire (HCCQ) measured the degree to which the patients experienced autonomy support from their HCPs [25]; the 21-item Treatment Self-Regulation Questionnaire (TSRQ) comprised three subscales measuring the patients’ motivations for taking diabetes medication, checking glucose levels, following their diets, and exercising regularly, wherein the results were scored as autonomous (originated from the self), controlled (pressured or coerced by intrapsychic or interpersonal forces), or amotivated (having no intention to change and often feeling unable to change) [26]; the 20-item Problem Areas In Diabetes (PAID) questionnaire, which is a five-point scale measuring the perceived burden of diabetes-related problems [27]; and the five-item World Health Organization-5 scale (WHO5) measured the adolescents’ emotional wellbeing [28].To capture how parents’ participation in GSD-Y might have impacted the adolescents’ perceptions of parental autonomy and involvement, two subscales from the Perception Of Parents Scale (POPS, a seven-point Likert scale) were chosen, consisting of 26 items (13 for mothers and 13 for fathers) [29]. All of the scales were consistent with the theoretical framework of GSD [30]. They were all available in Danish, except POPS, which was translated into Danish following the recommended guidelines [31].

In GSD-Y, the acquisition of life skills is considered to be a developmental process in which the adolescents start to accept and integrate diabetes into their lives and become autonomously motivated to handle the challenges that life as a teenager with type 1 diabetes demands [32]. Because part of developing life skills is making self-determined decisions, self-determination theory (SDT) plays a central role in GSD-Y. According to SDT, self-determined behavior requires an environment that is autonomy-supportive to foster competence, autonomy, and relatedness [32]. Signs of improved life skills were increases in scores on HCCQ, PCD, TSRQ autonomy, TSRQ relative autonomy index (formed by subtracting the TSRQ-scores on control from the TSRQ-scores on autonomy), and WHO5 (wellbeing) as well as decreases in scores on PAID, TSRQ control, and TSRQ amotivation. Increases in POPS for autonomy support and involvement were signs of increased life skills resulting from the parents’ participation.

Details of the scales and scores have been published elsewhere [13]. The face validity of all scales was tested in eight adolescents with type 1 diabetes (not included in the randomized part of the trial) before starting the trial; no changes were needed. The scales were compiled into one questionnaire and completed by the adolescents in the clinic at baseline, before randomization, at the end of the experimental period, and after a six-month follow-up period.

Secondary outcomes directly related to patient management that might be influenced by GSD-Y included the registration of: (1) insulin delivery (continuous subcutaneous insulin infusion (CSII) or multiple daily injections (MDI)), (2) the number of self-monitored blood glucose (SMBG) values during the prior (last) week, (3) hypoglycemic episodes (frequency and severity), and (4) admissions to the hospital as well as the reasons for the admissions (such as episodes of ketoacidosis or hypoglycemia).

Secondary outcomes indirectly related to patient management included a registration of (1) attendance at the intervention or control sessions, and (2) parental participation in adolescents’ visits and GSD-Y parent’s participation in their two GSD-Y visits.

The adolescents’ usual HCPs used a CRF to collect the direct and indirect secondary outcomes during the experimental period at every outpatient clinic visit, except for the number of SMBG measurements, which were self-reported when the adolescents completed the questionnaires. The number and severity of hypoglycemic episodes since the last visit were recorded, distinguishing between mild (treatable by the patient), moderate (requiring help from others), or severe (the patient was unable to assist in his or her own care, was semiconscious or unconscious, or was comatose) [33]. The plasma glucose levels at the time of the hypoglycemic episodes were unavailable. Demographic data were collected from adolescents at baseline.

To ensure that HCPs continued to correctly practice GSD-Y, fidelity was assessed by collecting a copy of the completed reflection sheets from all participants and 37 digital recordings from all HCPs during the trial. The recordings and reflection sheets were assessed by GRH.

### Statistical analysis

A power calculation based on an absolute difference of 11 mmol/mol (1.0%) in the primary outcome HbA1c between the GSD-Y and control groups, a standard deviation of HbA1c of 1.3% (as reported in a study on coping skills training [34]), a power of 0.80, and a two-tailed significance test at the 0.05 level, indicated that 26 patients would be needed in each group. To allow for 25% attrition, we aimed to recruit 68 adolescents.

Intention-to-treat analyses were used with two-tailed tests at the 0.05 significance level. Holm’s test was used to control the family-wise error rate [35].

The analyses of the primary outcome and each of the 14 continuous life skills outcomes were performed using a linear mixed model with repeated measures that assumed an unstructured covariance matrix. The primary result was based on a model that included an indicator for the intervention (I, reference group 2), an indicator for the follow-up (F, 0 for the end of the experiment and 1 for the follow-up time point), the interaction between the two indicators (I × F), the protocol-specified stratification variable, and the baseline value of the dependent variable Two hypotheses were tested: (1) that the intervention had an effect on the mean level of the dependent variable at the end of the experiment that was sustained until follow-up (main effect of I); and (2) that the intervention changed the level of the dependent variable from the end of the experiment until follow-up (interaction between intervention and follow-up). Thus, a significant main effect of the intervention in the presence of an insignificant main effect of follow-up and insignificant interaction between follow-up and intervention would suggest that the intervention had an immediate effect that was neither augmented nor blunted during the follow-up period. Two additional exploratory analyses were conducted: (1) an analysis without adjusting for the stratification variable (HCP), and (2) an analysis with an additional adjustment of the baseline value of log (HbA1c) to adjust for severity of the disease. The rate data were compared between the groups using a non-parametric Mann-Whitney U-test. The binary quantities were compared using the Cochran-Mantel-Haenszel test of relative risk. The ordinal data were compared between the groups using the Cochran-Armitage test for trends at the end of the experiment and at the end of the follow-up period. The mixed model with repeated measures utilizes all observed values and provides unbiased estimates under the condition that the data are missing at random (that the missingness of the data does not depend on the unmeasured values).

The fact that the HbA1c levels were measured relatively routinely in the adolescents allowed a supplementary *post hoc* analysis of the HbA1c levels, which was designed to investigate the constant periods and the frequencies of observation of the HbA1c levels. We compared the time series of the two groups, including the HbA1c level measurements obtained every third month, starting with the measurement obtained three months following randomization and covering a period of 30 months, to ensure that the period of experimental and control intervention was included for all patients. The results were subjected to a repeated-measures mixed-model regression analysis. The Akaike information criterion was used to choose between an autoregressive model of first order AR [1] and a compound symmetric covariance matrix because convergence was not obtained using an unstructured matrix. We tested for a main effect of intervention, a main effect of time, and an interaction between the two models, controlling for the baseline HbA1c level and the HCP. The data were analyzed using SPSS version 17 (IBM Corporation, 590 Madison Avenue, New York, NY 10022, USA) and SAS version 9.3(SAS Institute Inc. 100 SAS Campus drive, Cary, NC 27513-2412, USA).

## Results

**Figure 1:**
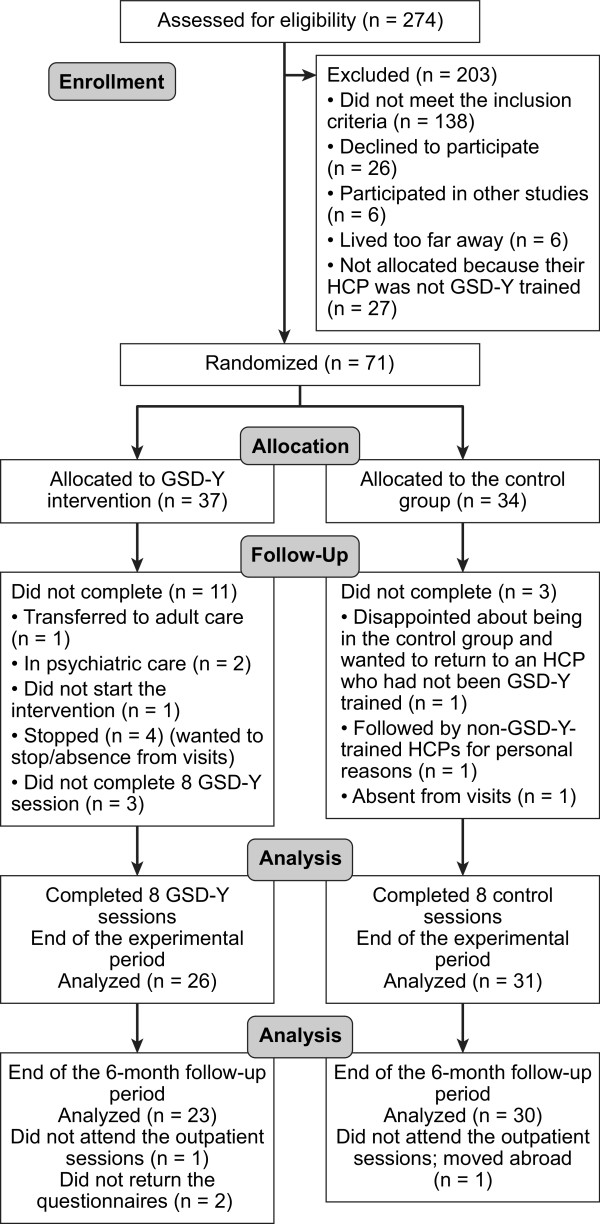
**Flow diagram of adolescents through the trial.** HCP: health care providers, GSD-Y: Guided Self-Determination-Youth, n: number.

A total of 71 of the 274 adolescents with type 1 diabetes were randomized to either the GSD-Y intervention group (n = 37) or the control group (n = 34; Figure 1). We allocated equal numbers of GSD-Y and control adolescents to each physician and nurse (approximately 10 adolescents to each professional). A total of 138 adolescents did not meet the eligibility criteria (first because of not meeting the HbA1c level criterion, second because of language barriers, and third because of current psychological or psychiatric treatment or possible psychiatric disorders). Twenty-seven eligible adolescents were not invited because they were usually treated by HCPs who had not been GSD-Y trained, 26 eligible adolescents declined to participate, six participated in other projects, and six lived far away and normally only attended the outpatient clinic three to four times per year.

A comparison of the baseline characteristics between the groups suggests that the randomization was successful for all variables except the HbA1c level, for which the mean value was somewhat lower in the control group (Table 1).

Fifty-seven adolescents (80%) completed the trial, 26 in the GSD-Y group and 31 in the control group. Fifty-three (75%) adolescents provided six-month follow-up data, 23 in the GSD-Y group and 30 in the control group. The duration of the experimental period was significantly longer in the GSD-Y group than in the control group (608 ± 125 days versus 458 ± 111 days, *P* <0.0005, mean ± SD). The duration of follow-up did not differ significantly between the groups (216 ± 59 days versus 246 ± 83 days, *P* = 0.14).

Each HCP completed the sessions with one to six adolescents from the GSD-Y group as well as between two to six adolescents from the control group.

### Primary outcome

The baseline HbA1c values were 80 ± 3 mmol/mol (9.5 ± 0.3%) in the GSD-Y group versus 73 ± 2 mmol/mol (8.8 ± 0.1%) in the control group (mean ± SE). At the end of the trial, the HbA1c levels were 80 ± 3 mmol/mol (9.5 ± 0.3%) in the GSD-Y group versus 76 ± 2 mmol/mol (9.1 ± 0.2%) in the control group. After a six-month follow-up, the results were 82 ± 3 mmol/mol (9.6 ± 0.3%) in the GSD-Y group versus 79 ± 3 mmol/mol (9.4 ± 0.3%) in the control group. The mixed-model analysis showed neither a significant main effect of the intervention (*P* = 0.85) nor any significant interaction between follow-up and intervention (*P* = 0.68). The main effect of follow-up and the interaction between the intervention and follow-up was clearly insignificant in all mixed-model analyses (the primary outcome and each of the 14 secondary outcomes). Thus, in all cases, the model was reduced to include the intervention indicator, the stratification variable (HCP), and the baseline value variable. Table 2 shows the group means with the 95% confidence interval, the difference between the mean in the two groups with the corresponding 95% confidence interval (CI), and the *P* value of the main effect.

**Table 2:** **Results of the mixed-model analyses with adjustment for effect of HCP and baseline values**

Outcome	Min – max score	GSD-Y	Control	Mean square difference between	95% CI of mean	*P* ^a^
		Mean square	Mean square	GSD-Y and control group	Difference	
HbA1c mmol/mol^b^		76.2	76.8	0.99	0.92-1.07	0.85
Blood sugar measurement numbers in one week		32.09	31.86	0.23	–6.23-6.69	0.94
PAID	0-100	27.52	26.58	0.934	–8.74-10.61	0.85
HCCQ	5-35	31.45	31.24	0.231	–1.51-1.94	0.81
PCD	5-35	26.39	27.70	–1.31	–3.93-1.30	0.32
TSRQ Autonomy	8-56	46.34	43.39	2.94	0.56-5.32	0.017
TSRQ Control	9-63	39.46	41.11	–1.65	–6.30-3.01	0.48
TSRQ Amotivation	4-28	8.28	11.50	- 3.22	–5.06-1.38	0.0013^c^
TSRQ Index	−51- + 47	6.87	2.02	4.85	0.80-8.89	0.020
POPS autonomy mother	7-49	39.11	35.57	3.54	0.24-6.84	0.036
POPS autonomy father	7-49	37.06	33.22	3.83	–0.29-7.96	0.068
POPS Involvement mother	6-42	34.83	33.06	1.74	–1.14-4.68	0.23
POPS Involvement father	6-42	31.13	29.2	1.199	–2.47-4.87	0.51
WHO5	0-100	57.99	60.0	–1.602	–10.32-7.11	0.71

^a^Because *P* of follow-up and *P* of project × follow-up were both >0.05, the analysis was repeated without the terms project and project × follow-up included in the model. The *P* value is that of the latter analysis.

^b^In the analysis, HbA1c was logarithmically transformed. The results have been transformed back to the original scale.

^c^ *P* <0.00128 (to preserve a family-wise error rate of less than 0.05, the significance level was adjusted to 0.00128 using Holm’s test).

HbA1c: Glycated Haemoglobin.

PAID: Problems Areas In Diabetes.

HCCQ: Health Care Climate Questionnaire.

PCD: Perceived Competence in Diabetes Scale.

TSRQ: Treatment Self-Regulation Questionnaire.

POPS: Perception of Parents Scale.

WHO5: World Health Organization-5scale.

**Figure 2:**
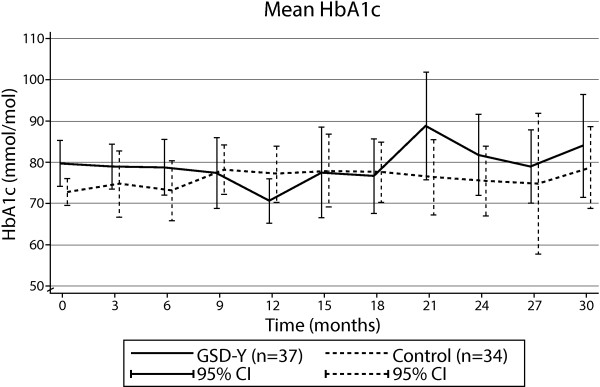
**HbA1c levels.** HbA1c levels in the GSD-Y and the control groups during 30 months of trial.

The mixed-model exploratory analysis of the time course of the HbA1c level during the first 30 months following the randomization revealed no significant main effect for the intervention (*P* = 0.86), no significant main effect for the time from randomization (referred to as time in the following) (*P* = 0.65), and no significant interaction between time and intervention (*P* = 0.55). Figure 2 shows the means plus/minus two standard deviations (SDs) and 95% confidence interval (CI) in each group as a function of time during the 30-month-period.

### Secondary outcomes

The results of the mixed-model analyses on the life skills scores controlling for baseline values and the effect of HCPs showed a significant main effect of the GSD-Y intervention on Treatment Self-Regulation Questionnaire (TSRQ) amotivation (*P* = 0.001), TSRQ autonomy (*P* = 0.017), TSRQ autonomy index (*P* = 0.020), and POPS autonomy mother support (*P* = 0.036; Table 2). After adjusting the significance level according to Holm’s method [35], only the main effect of the GSD-Y intervention on TSRQ amotivation (*P* = 0.0010) at the end of the intervention remained significant. The mean values of the life skills scores taken at baseline, at the end of the intervention, and at the end of the six-month follow-up period are presented in Table 3.

**Table 3:** **Results of the life skills questionnaires**

		Baseline		End of intervention		End of follow-up	
Quantity	Min-max scores	GSD-Y	CONTROL	GSD-Y	CONTROL	GSD-Y	CONTROL
PAID	0-100	29 ± 2.3 (34)	24 ± 3.1 (34)	28 ± 3.3 (26)	28 ± 4.0 (29)	26 ± 3.6 (22)	22 ± 3.5 (30)
HCCQ	5-35	31 ± 0.6 (37)	30 ± 0.9 (34)	32 ± 0.8 (26)	31 ± 0.6 (30)	32 ± 1.3 (23)	31 ± 1.1 (30)
PCD	5-35	24 ± 1.1 (37)	26 ± 1.0 (34)	26 ± 1.3 (26)	28 ± 0.9 (30)	28 ± 1.3 (23)	28 ± 1.3 (30)
TSRQ autonomy	8-56	45 ± 1.1 (37)	44 ± 1.3 (34)	47 ± 0.95 (26)	43 ± 1.3 (30)	46 ± 1.3 (23)	44 ± 1.3 (29)
TSRQ control	9-63	40 ± 1.4 (37)	41 ± 1.7 (34)	40 ± 1.5 (26)	41 ± 2.1 (30)	37 ± 2.3 (23)	40 ± 2.1 (29)
TSRQ amotivation #	4-28	11 ± 0.6 (37)	11 ± 0.6 (34)	9.1 ± 0.7 (26)	11 ± 0.9 (30)	8.6 ± 0.9 (23)	11 ± 0.8 (29)
TSRQ autonomy index (autonomy – control)	−51- + 47	4.8 ± 1.6 (37)	3.6 ± 1.3 (34)	6.9 ± 1.4 (26)	1.6 ± 1.3 (30)	9.0 ± 2.2 (23)	3.8 ± 2.1 (29)
POPS autonomy support mother	7-49	35 ± 1.0 (35)	35 ± 1.3 (34)	37 ± 1.5 (24)	35 ± 1.3 (24)	40 ± 1.2 (21)	37 ± 13 (29)
POPS autonomy support father	7-49	34 ± 1.5 (33)	33 ± 1.3 (32)	36 ± 1.8 (24)	34 ± 1.5 (24)	36 ± 2.1 (21)	33 ± 1.7 (29)
POPS involvement mother	6-42	33 ± 1.0 (34)	32 ± 0.8 (34)	34 ± 1.6 (24)	33 ± 1.2 (30)	36 ± 1.2 (21)	33 ± 1.1 (29)
POPS involvement father	6-42	31 ± 1.6 (32)	28 ± 1.1 (32)	31 ± 1.8 (24)	30 ± 1.4 (28)	32 ± 1.8 (21)	29 ± 1.4 (29)
WHO5 index	0-100	60 ± 2.8 (36)	66 ± 3.3 (34)	60 ± 4.2 (26)	61 ± 3.6 (30)	56 ± 4.8 (23)	62 ± 3.4 (30)

The results were taken at baseline, the end of intervention, and the end of the six-month follow-up period in the Guided Self-Determination-Youth group (GSD-Y) and in the treatment-as-usual control group. Data are presented as means ± standard errors (number of patients).#*P* = 0.0013 by mixed-model analysis; family-wise error controlled by Holm’s method [35].

PAID: Problems Areas In Diabetes.

HCCQ: Health Care Climate Questionnaire.

PCD: Perceived Competence in Diabetes Scale.

TSRQ: Treatment Self-Regulation Questionnaire.

POPS: Perception of Parents Scale.

WHO5: World Health Organization-5scale.

There were no significant differences between the GSD-Y group versus the control group concerning the number of SMBG measurements taken during the experimental period (32 ± 14 versus 32 ± 13 measurements per patient per week, *P* = 0.94) or at follow-up (31 ± 13 versus 31 ± 19, *P* = 0.88). The occurrence of mild, moderate, and severe hypoglycemic episodes during the experimental period were 0.60, 0.13, and 0.08 respectively, in the GSD-Y group (per patient per year) versus 2.4, 0.11, and 0.02 respectively, in the control group (per patient per year). No significant between-group differences were observed concerning the risk of hypoglycemia (mild: RR = 0.96, 95% CI 0.50 to 1.85, *P* = 0.91; moderate: RR = 2.31, 95% CI 0.46 to 11.6, *P* = 0.30; severe: RR = 2.3, 95% CI 0.46 to 11.6, *P* = 0.30) or the rate of events (mild: *P* = 0.80, moderate: *P* = 0.34, severe: *P* = 0.34) during the experimental period. Insulin regimens, insulin doses, admissions to hospital, and occurrences of ketoacidosis did not differ between the groups (data not shown).

The adolescents in the GSD-Y group needed more than one visit per session to complete the scheduled reflection sheets. The median number of visits was 12 (range: 8 to 16) in the experimental group compared with eight (range: 7 to 12) in the control group (*P* = 0.001). Neither the GSD-Y group patients nor the control group patients showed up for all scheduled outpatient sessions. The GSD-Y group had more non-attendance incidents yearly compared with the control group (0.9 ± 1.1 versus 0.4 ± 0.6 missed visits, *P* = 0.02), but the yearly number of cancellations did not differ between the two groups (1.1 ± 1.1 versus 0.8 ± 1.4 cancellations, *P* = 0.07). The parents of the GSD-Y adolescents participated in fewer sessions than the parents of the control adolescents (median: 3.5 versus 7 visits, *P* = 0.05). Twenty-three (68%) of the GSD-Y parents attended one parental GSD-Y session (at a median of six months; range: 2 to 14), and 11 parents (30%) attended two parental GSD-Y sessions (at a median of 13 months; range: 5 to 20).

More GSD-Y adolescents (50%) were referred to the dietician compared to the control group (11%). Each GSD-Y adolescent completed between one and six visits with the dietician, whereas each control adolescent had one visit.

The reflection sheets were completed by all 26 GSD-Y adolescents, except for two sheets identifying the patterns and motivations for blood sugar management behaviors (3.d and 4.a [13]), which were not used by 10 participants (39%).

## Discussion

When integrated into routine pediatric outpatient diabetes visits, GSD-Y had no significant effect on the primary outcome of HbA1c compared with treatment-as-usual. GSD-Y seemed to significantly decrease the level of amotivation for diabetes self-management at the end of the experimental period compared with the control group, an effect that was maintained at follow-up. No other life skills outcomes and no diabetes outcomes directly related to patient management were significantly influenced by the GSD-Y intervention compared with treatment-as-usual.

Our HbA1c results aligned with the results from three recently published randomized clinical trials that also included treatment-as-usual outpatient visits [9–11]. In the Development and Evaluation of a Psychosocial Intervention in Children and Teenagers Experiencing Diabetes (DEPICTED) trial (a diabetes training program for pediatric diabetes teams that is based on motivational interviewing) 26 secondary and tertiary care pediatric diabetes services in the UK were evaluated [9]. This intervention included 359 young people with type 1 diabetes (aged between 4 and 15 years) and their main caregivers. The program showed no effect on HbA1c levels one year after training compared with 334 patients in the control group. In the 18-month Families and Adolescents Communication and Teamwork Study (FACTS), the effectiveness of a family-centered group education program was studied in 158 adolescents with type 1 diabetes (aged between 11 and 16 years) [10]. Six 90-minute monthly sessions were attended by adolescents and parents. After 18 months (12 months post-intervention), there was no significant difference in the HbA1c levels compared with the 147 adolescents in the control group. In a two-year trial, Katz *et al*. randomized 153 adolescents (aged between 8 and 16 years) with type 1 diabetes into three groups: (1) receiving standard care, (2) receiving monthly outreach by a care ambassador, or (3) receiving monthly outreach by a care ambassador and participating in a family-focused psychoeducational intervention [11]. No significant differences in HbA1c levels were detected among the groups after two years.

In the DEPICTED trial, the HbA1c levels did in fact increase in both groups during the trial (from 79 to 83 mmol/mol (9.4 to 9.7%)) in the intervention group and from 77 to 80 mmol/mol (9.2 to 9.5%) in the control group) [9], and similar findings were observed in FACTS [10] and the trial by Katz *et al*. [11]. In our trial, the HbA1c levels increased in the control group (from 73 to 76 mmol/mol [8.8 to 9.1%]) at the end of the experimental period) but were unchanged in the GSD-Y group (80 mmol/mol, 9.5%) from baseline until the end of the experimental period. It is well known that HbA1c levels normally increase during adolescence [36]. In the DanDiabKids Registry, the HbA1c levels increased from 66 to 73 mmol/mol (8.2 to 8.8%) in adolescents with type 1 diabetes who were between the ages of 12 and 18 years, or at an average of 1 mmol/mol (0.12%) per year (Svensson J, unpublished data 2012). Whether our finding of an unchanged average HbA1c level in the GSD-Y group during the trial period represents a true difference from the increase in the control group or is a coincidence remains to be determined.

In adults, the original 16-hour, nurse-led GSD group training had a statistically significant impact on HbA1c levels from 3 to 12 months [12]. Group interventions in adolescents have been found to be associated with improved glycemic control compared to individual interventions [8, 34, 37]. The lack of an effect of GSD-Y on HbA1c in our study could therefore be attributed to our individual approach. However, in FACTS, poor attendance at group education sessions delivered in a routine clinic was a major challenge [10]. The authors suggested that more personalized educational approaches might be required to support and motivate families struggling to integrate the demands of intensive insulin regimens into their daily lives [10], a statement that seems to be somewhat contradicted by our findings that GSD-Y is a personalized, motivating approach. The non-significant results of the four trials ([9–11] and the present trial) appear to be related to the more complex conditions that are at play among adolescents compared with adults. Adolescents’ crave conformity; that fact, their lack of acceptance of the disease [38], and their perception of resistance against their parents [39] are important factors to consider in achieving good glucose management. These competing difficulties may have resulted in less attention paid to the reflection sheets. One-third of the GSD-Y participants did not complete one or two of these reflection sheets, which were designed to identify the adolescents’ patterns of motivation for blood sugar management [12]. These reflection sheets may be too difficult or demanding for some adolescents because adults filled in all of the reflection sheets [30]. Another distinction from the trial in adults was that the GSD-Y adolescents required more time and additional visits to complete the eight GSD-Y sessions and had a higher rate of non-attendance than the control group. We speculate that the extended time between the GSD-Y sessions and an excessively lengthy intervention period may have reduced the momentum of the intervention to impact glucose management behavior [40]. This consideration is also mentioned in the report from Katz *et al*. [11], yet, it is also possible that a reduction in the HbA1c in adolescents is not achievable through this GSD-Y version.

GSD-Y significantly decreased amotivation at the end of the intervention period compared with treatment-as-usual. This significant main effect of the intervention in the presence of an insignificant main effect of follow-up and insignificant interaction between follow-up and intervention suggests that the intervention had an immediate effect that was neither augmented nor blunted during the follow-up period. This result may seem paradoxical considering the aforementioned difficulties in complying with the intervention. A decrease in amotivation for taking insulin, checking blood sugar, and following diet and exercise regimes regularly indicates that GSD-Y adolescents began a process of becoming more engaged in their own diabetes management [26]. This is an important sign of developing life skills [21]. As described by Levesque *et al*. [41], people lack motivation when they are amotivated and are therefore not self-determined [32]. Moreover, people fail to behave in a purposeful manner and ‘experience no meaningful relation between what they are doing and themselves’ ([41] p. 692). Such a way of acting is inevitably half-hearted and connected with a sense of feeling helpless and expecting failure [32] ‘I do not know why I do try - I will not be successful’[13 p. 9]. Amotivation has been regarded as a sign of hopelessness and a predictor of psychological distress and depression [42]. Decreasing amotivation seems thus to be important for a constructive approach to diabetes self-management [43]. The decreased amotivation was not found to be accompanied by significantly increased autonomy support from parents (POPS) or HCPs (HCCQ). Parents and HCPs may, however, unwittingly have previously contributed to decreasing the adolescents’ motivation for treating the disease and instead foster resistance, passivity, and amotivation for developing self-management skills [44–46]. One explanation for the fact that the decrease in amotivation was the only significant change may be that decreasing amotivation is a kind of turning point, as described by Hernandez [47], and a first step in becoming more engaged in one’s own diabetes care. In a qualitative evaluation of GSD-Y [48], adolescents, HCPs, and parents valued the reflection sheets as important in engaging the adolescents and giving them a voice in their relationships with their HCPs. The decreased amotivation may also have been because the GSD-Y adolescents received more visits during a longer time period. However, none of the other life skills outcomes were influenced by the time differences between the intervention and the control group.

Our study demonstrated no significant effects on the remaining life skills parameters when Holm’s correction [35] was implemented. The lack of significant differences in the scales between the groups may have been due to the sample size. Accordingly, type II errors cannot be excluded.

The present trial has several strengths. First, we used stratified randomization, which reduced selection bias by ensuring that GSD-Y and control adolescents were followed by equally GSD-Y-skilled HCPs. Second, we chose the same primary and secondary outcomes that were used in adults precisely because they had been proven sensitive to capturing the effects of GSD in adults [12]. Thus, it was possible to test whether an effect occurred in adolescents. Third, we assessed the HCPs’ fidelity in correctly delivering GSD-Y during the trial by reviewing the completed reflection sheets and digitally recording the outpatient session. However, the feasibility of integrating a complex intervention in a complex healthcare system may be questioned because the participants followed the protocol in neither the experimental nor the control group. A pilot study might have captured some of the difficulties involved in integrating GSD-Y into usual outpatient visits [49], but we did not choose to do so because we would have been left with too few adolescents for the randomized trial.

Several limitations may have threatened the internal and, hence, the external validity of our trial. First, we achieved allocation concealment by employing opaquely sealed envelopes [50, 51]. Although they were consecutively numbered, we cannot exclude the possibility that the allocation sequence was compromised [50, 51]. When the expected adolescents did not show up as scheduled or needed time to consider their participation until the following visit, the next adolescent who fulfilled the inclusion criteria was invited and randomized if his or her consent to participate was given. Second, the present trial could not be blinded because of the nature of the intervention, which may have biased our results [50, 51]. Moreover, because each HCP practiced both the experimental and the control intervention, we cannot exclude a spillover effect caused by the GSD-Y training of all HCPs. Third, we did not assess some of the secondary outcomes directly related to patient management during the follow-up period. The GSD-Y impact on, for instance, the occurrence and the risks of hypoglycemia could, therefore, only be assessed during the experimental period. Furthermore, it may also be considered a limitation that HbA1c was chosen as the primary outcome both at the end of the experimental period and during follow-up because the time of the experimental period differed significantly between the two intervention groups. HbA1c is, however, considered to be the ‘gold standard’ when researching outcomes in adolescents with type 1 diabetes as an indicator of diabetes management [52, 53]. To compare our results with similar trials, we chose this outcome variable. However, no effect of the experimental intervention was detected by using HbA1c in our trial. Finally, one study limitation is that 11 adolescents from the GSD-Y group did not complete the intervention. This may indicate that the intervention was too demanding for some adolescents or that the use of ‘pen and paper’ for the reflection sheets is in contrast with the contemporary youth’s typical communication media. We also wonder whether the major difference between HCPs in helping GSD-Y adolescents may depend on the individual skills of HCPs.

## Conclusions

No effect of GSD-Y on HbA1c was identified in our trial. Our results can be questioned because the intervention was not followed as strictly as was intended. Together with previous research [9–11], the result underscores the difficulties involved in developing effective treatments integrated into the usual care provided to adolescents. Presently, GSD-Y should not be integrated into outpatient visits in its current format if the only purpose is to improve glycemic control. Whether the positive finding of decreased amotivation in the GSD-Y group can be sustained for longer periods or replicated remains to be determined.

## Abbreviations

BAE: Bente Appel Esbensen
BT: Birger Thorsteinsson
CG: Christian Gluud
CRF: Case Report Form
CSII: Continuous subcutaneous insulin infusion
DanDiabKids: Danish National Diabetes Register for Children
EH: Eva Hommel
HbA1c: Glycated Haemoglobin A1c (a difference of 1% is equivalent to 11 mmol/mol)
HCCQ: Health Care Climate Questionnaire
HCP: Healthcare providers
GRH: Gitte Reventlov Husted
GSD: Guided Self-Determination
GSD-Y: Guided Self-Determination-Youth
MDI: Multiple Daily Injections
PW: Per Winkel
PAID: Problems Area In Diabetes
PAR: Participatory Action Research
PCD: Perceived Competence in Diabetes
POPS: Perception Of Parents Scale
SDT: Self-Determination theory
SMBG: Self-monitored blood glucose measurements
TSRQ: Treatment Self-Regulation Questionnaire
VZ: Vibeke Zoffmann
WHO5: Emotional Well-Being Scale.
